# Socioeconomic position and ten-year survival and virologic outcomes in a Ugandan HIV cohort receiving antiretroviral therapy

**DOI:** 10.1371/journal.pone.0189055

**Published:** 2017-12-15

**Authors:** Andrew G. Flynn, Godwin Anguzu, Frank Mubiru, Agnes N. Kiragga, Moses Kamya, David B. Meya, David R. Boulware, Andrew Kambugu, Barbara C. Castelnuovo

**Affiliations:** 1 Infectious Diseases Institute, Kampala, Uganda; 2 School of Medicine, Makerere University College of Health Sciences, Kampala, Uganda; 3 Department of Medicine, University of Minnesota, Minneapolis, Minnesota, United States of America; Azienda Ospedaliera Universitaria di Perugia, ITALY

## Abstract

Lifelong ART is essential to reducing HIV mortality and ending the epidemic, however the interplay between socioeconomic position and long-term outcomes of HIV-infected persons receiving antiretroviral therapy (ART) in sub-Saharan Africa is unknown. Furthering the understanding of factors related to long-term ART outcomes in this important region will aid the successful scale-up of ART programs. We enrolled 559 HIV-infected Ugandan adults starting ART in 2004–2005 at the Infectious Diseases Institute in Kampala, Uganda and followed them for 10 years. We documented baseline employment status, regular household income, education level, housing description, physical ability, and CD4 count. Viral load was measured every six months. Proportional hazard regression tested for associations between baseline characteristics and 1) mortality, 2) virologic failure, and 3) mortality or virologic failure as a composite outcome. Over ten years 23% (n = 127) of participants died, 6% (n = 31) were lost-to-follow-up and 23% (107/472) experienced virologic treatment failure. In Kaplan-Meier analysis we observed an association between employment and mortality, with the highest cumulative probability of death occurring in unemployed individuals. In univariate analysis unemployment and disease severity were associated with mortality, but in multivariable analysis the only association with mortality was disease severity. We observed an association between higher household income and an increased incidence of both virologic failure and the combined outcome, and an association between self-employment and lower incidence of virologic failure and the combined outcome when compared to unemployment. Formal education level and housing status were unrelated to outcomes. It is feasible to achieve good ten-year survival, retention-in-care, and viral suppression in a socioeconomically diverse population in a resource-limited setting. Unemployment appears to be related to adverse 10-year ART outcomes. A low level of formal education does not appear to be a barrier to successful long-term ART.

## Introduction

Socioeconomic position—an aggregate of resources including education, employment, income and wealth that interact but are not interchangeable—is linked to health outcomes in a variety of diseases and settings.[1–4] Among HIV-infected individuals receiving antiretroviral therapy (ART) in high-income countries, less wealth, income or formal education conveys risk for poorer survival.[5–11] Similarly, food insecurity, less formal education and homelessness conveys risk for decreased viral suppression in high-income countries.[5, 12, 13] However, although HIV/AIDS is the leading cause of death in sub-Saharan Africa and the number of individuals receiving ART is increasing,[14, 15] any relationship between socioeconomic position and ART outcomes in this important region is poorly understood.

Scale-up of ART and viral load monitoring in sub-Saharan Africa is recent and limited[15] and, as a result, most literature on socioeconomic determinants examines pre-ART outcomes. Regional literature indicates higher socioeconomic position may convey an increased risk of HIV infection among the general population and, among HIV-infected individuals in care before starting ART, lower socioeconomic position may convey an increased risk of loss-to-follow-up and death.[16–25] The limited literature examining socioeconomic position and ART outcomes in Africa is plagued by short follow-up times. Studies report a higher relative one-year mortality among ART patients with less wealth, no regular income, no formal educations, and unemployment,[26, 27] and in Uganda unemployment has been associated with two-fold higher four-year mortality on ART.[28] These African studies did not account for physical ability and disability, which is related to disease progression and death in people living with HIV, and may also be related to socioeconomic position.[29] Despite the fact that viral monitoring is the most objective measure of ART adherence and subsequent treatment success, regional literature examining socioeconomic position and viral suppression is limited to one 2003 South African study which reported no association between socioeconomic position and one-year viral suppression when ART was provided free of charge.[30]

Despite an incomplete understanding of the interplay between socioeconomic position and ART outcomes, a myriad of interventions have been proposed and implemented by NGOs and even ministries of health in sub-Saharan Africa to address financial and social barriers to successful ART.[31, 32] Furthering the understanding the complicated relationship between socioeconomic position and outcomes of HIV is crucial for these programs to complement ART and viral load scale-up and achieve the best possible outcomes. Our study, conducted at a free ART clinic in Kampala, Uganda, provides Africa’s first rigorous long-term assessment of the associations between different social and economic factors and the risk of mortality and virologic failure after ART initiation in a large, socioeconomically diverse, observational HIV cohort with low loss-to-follow-up and regular viral load testing. As successful lifelong ART is the cornerstone of strategies to reduce HIV mortality and end the HIV epidemic, understanding the factors related to long-term ART outcomes is essential.

## Methods

This prospective cohort study enrolled HIV-infected ART-naïve Ugandan adults ≥18 years of age who initiated ART between April 2004 and April 2005 at the Infectious Disease Institute clinic in Kampala, Uganda. Participants were followed for ten years. Eligibility criteria included at least two previous clinic visits, stable residence within 20 kilometers of Kampala, CD4 count <200 cells/μL or WHO stage IV disease, and written informed consent. At enrollment, we assessed HIV progression with baseline CD4 count, and we measured disability with the Karnofsky performance scale, which predicts mortality in HIV-infected persons.[29] A Karnofsky score <80 indicates difficulty with physical tasks.[33] The first-line ART regimen, initiated upon enrollment in the cohort, was stavudine or zidovudine plus lamivudine and either nevirapine or efavirenz. The study was approved by the Makerere University Ethics Committee and the Uganda National Council for Science and Technology Ethics Committee and written consent was obtained from each participant. More detailed descriptions of study procedures and data collection have been previously reported.[34, 35]

### Measuring socioeconomic position

At cohort enrollment, we interviewed participants to record baseline socioeconomic characteristics. Self-reported monthly household income was converted to USD and categorized as above or below 1 USD/day. We assessed: 1) highest level of schooling completed (primary or below, any secondary, or any post-secondary); 2) household income (no regular income, regular income below the 2004 international poverty line of 1 USD/day, and regular income above 1 USD/day); 3) employment status (unemployed, self-employed, employed by government/organization and employed by a private company) and 4) housing description (brick or mud). Self-employment was defined as the state of working for oneself, such as growing/creating products to sell or owning a small business, without an employer.

### Ascertainment of clinical outcomes

Clinic visits were scheduled every 6 months and participants were classified as lost to follow-up if they missed a clinic visit and were unreachable by telephone and home visit. Deaths were recorded and confirmed through medical record review and interviews with participants’ next of kin. HIV-1 viral load was measured in all participants at enrollment and afterwards every 6 months (Amplicor HIV-1 Monitor PCR Test version 1.5 with a detection limit of 400 copies/mL through 2011 and, more recently, using COBAS Ampliprep/COBAS Taqman HIV-1 Test Ver.2.0,Roche Diagnostics, Indianapolis, IN). Virologic treatment failure was defined as either lack of initial virologic suppression (at least one measurement below 400 copies/mL after ART initiation) or, after achieving initial suppression, two consecutive measurements >1000 copies/mL or one measurement >5000 copies/mL among patients who had no follow up measurement.[36, 37]

### Statistical analysis

We described baseline cohort clinical and socioeconomic characteristics stratified by sex. For categorical variables we compared proportions using the chi-square test and for continuous variables we compared medians using the Kruskal-Wallis test. We used Kaplan-Meier survival methods to calculate survival time and time to treatment failure stratified by baseline socioeconomic characteristics. Patients who died were followed up to known date of death or date of last clinic visit if death date unknown. Patients who were lost-to-follow-up were censored on last visit date, while all patients alive at ten years were administratively censored on the date of their ten-year clinic visit. For treatment failure, follow-up time began at the month six clinic visit or the first date of viral suppression and was calculated until the date of viral failure among those who failed. Among patients who died, who were lost-to-follow-up or who completed ten years of ART without failure, follow-up time was calculated until the date of death, the last visit seen or the ten-year clinic visit, respectively.

To estimate the predictive associations between socioeconomic variables and mortality and virologic treatment failure, we performed Cox proportional hazard regression for each outcome (1) mortality, 2) treatment failure and 3) composite outcome of mortality and treatment failure) including demographic and socioeconomic variables as well as baseline and time-varying CD4 count and functional impairment (Karnofsky score). We developed a multivariable model that included age, gender and other variables that reached *p* ≤0.25 in univariate analysis. Robust standard errors were used to account for within-patient correlation. Analysis was performed using Stata version 12 (Stata Corp, College Station, Texas).

## Results

We enrolled a total of 559 adults starting ART between April 2004 and April 2005. Demographic, socioeconomic and clinical characteristics at enrollment separated by gender are presented in Table 1. Median age was 35 years (IQR 30–41), 69% (n = 386) were women, and women participants were younger than men (*p* = 0.001). At baseline, 49% (n = 272) were unemployed, 46% (n = 257) had a primary school education or less, 68% (n = 378) reported no regular household income or income below the poverty line of USD <1/day, and 33% (n = 187) lived in a mud house. Women had lower socioeconomic indicators than men (*p*<0.001) except in housing, which did not differ (*p* = 0.32). Of 553 participants with CD4 count measured at enrollment, 53% (n = 292) had a CD4 count <100 cells/μL. Participants with CD4<100 were more likely unemployed (55%) than those with higher CD4 >100 cells/μL (42%, *p* = 0.04). At baseline, 34% (189/559) were functionally impaired with a Karnofsky performance scale <80. Compared to participants with no functional impairment, a larger proportion of those with a Karnofsky <80 were unemployed (70% vs 38%, *p*<0.01) and receiving no regular income or any income <1 USD/day (81% vs 61%, *p* <0.01). After ten years, 6% (n = 31) of participants were lost to follow-up. In Kaplan-Meier analysis, sex and employment status were associated with loss to follow-up, with men and unemployed individuals most likely to be lost from the cohort.

**Table 1:** Cohort descriptive characteristics.

Characteristic	Category	All Participants N = 559	Women N = 386	Men N = 173	*P* value
**Age (years)**	< 30	127 (23%)	104 (27%)	23 (13%)	
	30–40	280 (50%)	189 (49%)	91 (53%)	
	> 40	152 (27%)	93 (24%)	59 (34%)	
	Median (IQR)	35 (30–41)	33 (29–40)	38 (33–42)	
					0.001
**Formal education** **level**	None or any primary	257 (46%)	197(51%)	60 (35%)	
** **	Any secondary	232 (42%)	153 (40%)	79 (46%)	
** **	Any post-secondary	70 (13%)	36 (9%)	34 (19%)	
** **					<0.001
**Employment**	Unemployed	272 (49%)	204 (53%)	68 (39%)	
** **	Self-employed	142 (25%)	103 (27%)	39 (23%)	
** **	Gov't or organization	77 (14%)	38 (10%)	30 (17%)	
** **	Private employer	68 (12%)	41 (10%)	36 (21%)	
** **					<0.001
**Household income**	No income	184 (33%)	136 (35%)	48 (28%)	
** **	Income >1 US$/day	194 (35%)	156 (40%)	38 (22%)	
** **	Income < 1 US$/day	181 (32%)	94 (24%)	87 (50%)	
** **					<0.001
**Housing material**	Mud	187 (33%)	124 (32%)	63 (36%)	
** **	Brick	372 (67%)	262 (68%)	110 (64%)	
** **					0.32
CD4 count*	< 100	292 (53%)	196 (51%)	96 (56%)	
** **	100–200	185 (33%)	132 (35%)	53 (31%)	
** **	> 200	76 (14%)	53 (14%)	23 (13%)	
	Median (IQR)	98 (21–163	100 (29–170)	87 (13–152)	
** **					0.09
**Physical functional** **ability**	Impaired (Karnofsky <80)	189 (34%)	126 (33%)	63 (36%)	
** **	Not impaired	370 (66%)	260 (67%)	110 (64%)	
** **					0.38
**10-year mortality**	Dead	127 (23%)	86 (22%)	41 (24%)	
	In care or left cohort	432 (77%)	300 (78%)	132 (76%)	
					0.71
**10-year viral failureª**	Viral failure	107 (29%)	75 (30%)	32 (28%)	
	No failure	365 (71%)	250 (70%)	115 (72%)	
					0.81

Values are N(%) or median (interquartile range). *P*-value testing for gender differences in characteristics was done using the chi-square test

*Baseline CD4 count was available for 553 participants

ª10-year viral failure was available for 472 participants

### Mortality

After ten years, 72% (401/559) of participants were alive and in care at our clinic and 23% (n = 127) had died with 63% (n = 80) of deaths in the first year of HIV therapy. Kaplan-Meier survival analysis showed higher cumulative probability of death among participants with baseline CD4<100 cells/μL (*p*<0.01), among participants with Karnofsky <80 indicating baseline functional impairment (*p*<0.01), and among participants who were unemployed at enrollment (*p*<0.01). In the univariate proportional hazard regression age >40 years compared to a reference age <30 was associated with poorer survival, while Karnofsky score >80 and CD4 100–200 cells/μL compared with <100 were associated with improved survival (Table A in S1 File). Self employment or employment by government or an organization compared with unemployment, Karnofsky score >80 and CD4 of 100–200 cells/μL compared to <100 were all associated with improved survival. In the multivariable proportional hazard regression, compared to a reference age of <30 years, age between 30 and 40 years and >40 years was significantly associated with death. Karnofsky performance score >80 and baseline CD4 count of 100–200 cells/μL compared to CD4 <100 cells/μL were significantly associated with lower risk of mortality. No socioeconomic characteristics were significantly related to mortality in the multivariable model.

### Virologic treatment failure

Of the initial 559 participants, 84% (n = 472) were still alive and had at least one viral load measurement after 6 months of ART. Over the ten year period 23% (107/472) met the definition of virologic treatment failure, including 7% (33/472) who never achieved viral suppression during the first 24 months after initiating ART. In the univariate proportional hazard regression, we observed no significant association between any baseline characteristics and virologic failure (Table B in S1 File). In the multivariable proportional hazard regression, lower risk of virologic failure was significantly associated with age >40 years and self-employment compared to unemployment (hazard ratio, 0.60; 95%CI, 0.37–0.98). Any regular household income <1 USD/day (hazard ratio, 1.81; 95% CI 1.10–2.98) and regular income >1 USD/day (hazard ratio, 2.33; 95%CI 1.24–4.39) were both associated with higher risk of treatment failure compared to participants with no regular household income in multivariable analysis.

### Composite outcome (mortality or virologic failure)

**Fig 1:**
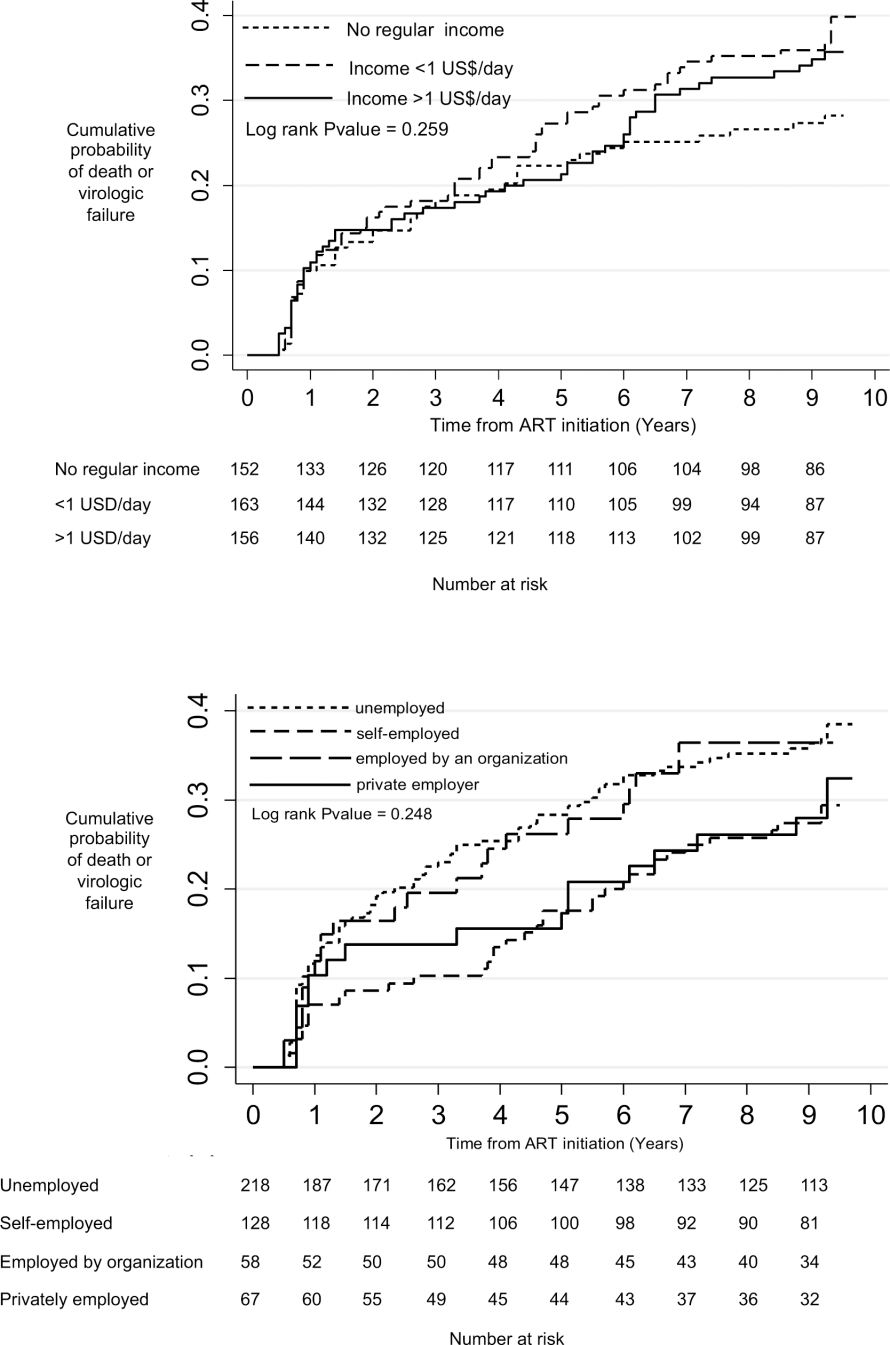
Death or virologic failure among HIV-infected participants surviving more than six months on ART grouped by household income and by participant employment status (N = 472). Caption: Fig 1A shows a trend towards increased risk of death and viral failure among participants with higher regular household income, while Fig 1B shows a trend towards decreased risk of death and viral failure among self-employed and privately employed participants. The multivariable proportional hazard regression identified a significant positive association between higher household income and higher incidence of the composite outcome and a significant association between self-employment and decreased incidence of the composit outcome compared with unemployment.

In Kaplan-Meier survival analysis cumulative probability of death or treatment failure was not significantly different when participants were grouped by baseline characteristics (Fig 1). When viewing death or viral failure together as a composite outcome in the univariate proportional hazard regression, Karnofsky score >80 was significantly associated with lower risk of the combined outcome (Table 2). In the multivariable regression, the composite outcome was significantly positively associated with any regular household income <1 USD/day (hazard ratio, 1.88; 95%CI 1.23–2.87)and with regular household income >1 USD/day (hazard ratio, 2.18; 95%CI 1.27–3.74) compared with no regular household income. Compared to unemployment, self-employment was significantly associated with decreased risk of the composite outcome of death or viral failure (hazard ratio, 0.59; 95%CI 0.38–0.92). To account for the possibility that participants lost to follow-up were not missing at random, we repeated the Cox regression with loss to follow-up included in the composite outcome. We did not observe any significant differences in the unadjusted or adjusted results.

**Table 2:** Risk factors for 10-year mortality or virologic failure (combined outcome) in a Ugandan cohort with baseline AIDS receiving ART (N = 472).

Baseline Category	Baseline Characteristics	Unadjusted Hazard Ratio (95% CI)	*P* value	Adjusted Hazard Ratio (95% CI)*	*P* value
**Sex**	Men	0.95 (0.67–1.34)	0.76	0.82 (0.56–1.20)	0.30
**Age, years**	< 30	1.00	Ref	1.00	Ref
	30–40	0.73 (0.49–1.08)	0.11	0.80 (0.53–1.21)	0.29
	> 40	0.97 (0.64–1.48)	0.90	0.95 (0.61–1.48)	0.82
**Highest education level**	Primary	1.00	Ref	1.00	Ref
	Secondary	1.22 (0.88–1.70)	0.24	1.24 (0.87–1.76)	0.23
	Tertiary	0.81 (0.45–1.43)	0.46	0.76 (0.40–1.45)	0.40
**Employment status**	Unemployed	1.00	Ref	1.00	Ref
	Self-employed	0.70 (0.47–1.03)	0.07	0.59 (0.38–0.92)	0.02
	Organization/Govt	0.97 (0.61–1.54)	0.89	0.83 (0.45–1.53)	0.56
	Privately employed	0.73 (0.43–1.23)	0.24	0.58 (0.31–1.09)	0.09
**Household income**	No regular income	1.00	Ref	1.00	Ref
	Income <1 US$/day	1.38 (0.93–2.06)	0.11	1.88 (1.23–2.87)	<0.01
	Income >1 US$/day	1.27 (0.85–1.91)	0.24	2.18 (1.27–3.74)	<0.01
**Physical housing**	Brick house	1.00	Ref		
	Mud house	1.00 (0.72–1.40)	0.99		
**Karnofsky Score**	Karnofsky ≥80	0.67 (0.48–0.93)	0.02	0.74 (0.52–1.05)	0.09
**CD4 count/μL**	<100	1.00	Ref		
	100–200	0.79 (0.55–1.12)	0.18	1.02 (0.70–1.48)	0.94
	>200	0.86 (0.54–1.37)	0.52	1.07 (0.65–1.75)	0.79
**Time-varying characteristics**	Karnofsky ≥80	0.10 (0.06–0.17)	<0.001	0.52 (0.42–0.65)	<0.001
	CD4 <100	1.00	Ref	1.00	Ref
	CD4 100–200	0.31 (0.19–0.52)	<0.001	0.50 (0.39–0.64)	<0.001
	CD4 >200	0.09 (0.05–0.15)	<0.001	0.36 (0.29–0.56)	<0.001

Hazard Ratio calculated via Cox proportional hazard model and *adjusted for sex, age, education, employment, income, baseline and time-varying Karnofsky and CD4

## Discussion

Our study is among the first to examine socioeconomic position and long-term ART outcomes in Africa. Employment and household income may be related to ten-year survival and viral suppression among individuals receiving ART in this setting. Employment status was found to be significantly associated with cumulative probability of death in Kaplan-Meier analysis, with unemployed participants experiencing death at the highest rate. Subsequently a clear significant relationship was identified between self-employment and lower incidence of the combined death and viral failure outcome when compared to unemployment in the adjusted Cox regression, after controlling for known confounding covariates such as physical disability. Although this data and methodology cannot ascertain causality, these observations suggest that unemployment may be a risk factor for long-term adverse ART outcomes, which supports similar findings in the literature on shorter ART outcomes in sub-Saharan Africa.[27, 38]

In our cohort data, household income was largely separate from employment status because it measured the contributions of all household members. Any association between household income and the mortality/viral failure endpoint was not strong enough to reach significance in Kaplan-Meier analysis or unadjusted Cox regression. However, clear significant relationships were identified in the adjusted model between higher household income and higher incidence of the composite outcome (death or viral failure). The measure of higher household income, separate from employment status, may have captured a risk factor related to decreased adherence and poor outcomes. Of note, the cohort subset with income >1USD/day, which exhibited the highest relative rate of viral failure, also had the highest self-reported medication adherence. Further research is needed to better understand the interplay between household income and ART outcomes.

Substantial variation in the cohort’s socioeconomic characteristics provided enough diversity to test for outcome differences. The long follow-up period with rigorous outcome ascertainment and low loss-to-follow-up of 6% also contribute to the results’ validity. Our inclusion of baseline CD4 count and functional impairment in the analysis enabled us to avoid the potential confounding of clinical status at the time of ART initiation. The different associations observed between various socioeconomic characteristics and outcomes reinforces the finding that socioeconomic position consists of separate factors that have distinct relationships with health and healthcare. Of note, formal education level was found to be unrelated to ten-year incidence of mortality or virologic failure. This finding is encouraging to HIV providers and ART programs serving populations with low levels of formal education. The ratio of women to men in this cohort represented the ratio of women and men enrolled at the Infectious Diseases Institute and in the country, likely due to higher HIV prevalence in women and more HIV screening in women, especially during antenatal care. We observed significant socioeconomic differences between women and men. However, we found no significant association between sex and any outcome, leading to the conclusion that sex alone was not predictive of survival or viral failure.

Existing literature widely encourages socioeconomic interventions for HIV patients [5, 26, 31, 39] and some HIV programs have implemented such interventions.[32] Our findings suggest efforts to boost employment among people living with HIV and receiving ART may promote successful long-term disease control. The Infectious Diseases Institute clinic provided routine support to all patients in this cohort, including counseling, telephone calls to schedule visits and after missed visits and one-on-one provider-patient encounters. Financial support for transport—a leading barrier to patient retention in Uganda [40]—was given four times per year with quarterly disbursements between USD 2.00 and 2.60. Ten-year viral suppression in our Ugandan cohort was similar to high-income countries [41] and should be encouraging to policymakers and program administrators seeking to reduce mortality and achieve viral suppression in socioeconomically diverse HIV-infected populations in low-income countries.

Several limitations of our data collection and analysis are worth mentioning and merit further research Although we used broad categories to measure socioeconomic variables, limitations exist with patient self-reported information. Our baseline measurement of socioeconomic position upon enrollment is a common method and may be sufficient for assessing relatively static characteristics such as education and housing material in adults.[6, 9, 28] However, the single measurement represents a significant limitation and further research may benefit from a time-varying measurement of employment and income as they change during years of treatment. Our measurements of household income placed participants in only three categories and did not account for household size or for barriers affecting adherence, such as social stigma and work or schedule barriers to regular ART that may help to explain an association between higher household income and poor outcomes. Although our measure of functional impairment with the Karnofsky performance status test adds an important component to our analysis, the Karnofsky scale is relative blunt tool. Further research may benefit from more detailed measures of cognitive or physical impairment. Kaplan-Meier and univariate proportional hazard regression methods of testing for predictive associations between socioeconomic characteristics and outcomes allow for straightforward interpretation of coefficients, although our data only allows tests for association, not causality. These adjusted hazard ratios provide additional information but must be interpreted with caution. Lastly, the resources available to our clinic were more than those available to most public sector clinics in low-income countries. Without sufficient clinic resources, lower socioeconomic position may emerge as a more prominent contributor to poor outcomes of patients on ART.

## Supporting information

S1 File Table A. Risk factors for all-cause 10-year mortality in a Ugandan cohort with baseline AIDS receiving ART (N = 559). Table B. Risk factors for 10-year virologic treatment failure in a Ugandan cohort with baseline AIDS receiving ART (N = 472).
(DOCX)

## References

[1] Rasmussen JN, Rasmussen S, Gislason GH, Buch P, Abildstrom SZ, Kober L, et al Mortality after acute myocardial infarction according to income and education. J Epidemiol Community Health. 2006;60(4):351–6. doi: 10.1136/jech.200X.040972 ; PubMed Central PMCID: PMCPMC2566173. 16537354 10.1136/jech.200X.040972 PMC2566173

[2] Marmot MG. Status syndrome: a challenge to medicine. JAMA. 2006;295(11):1304–7. doi: 10.1001/jama.295.11.1304 . 16537740 10.1001/jama.295.11.1304

[3] Phelan JC, Link BG, Diez-Roux A, Kawachi I, Levin B. "Fundamental Causes" of Social Inequalities in Mortality: A Test of the Theory. Journal of Health and Social Behavior. 2004;45(3):265–85. doi: 10.1177/002214650404500303 15595507 10.1177/002214650404500303

[4] Krieger N, Williams DR, Moss NE. Measuring social class in US public health research: concepts, methodologies, and guidelines. Annu Rev Public Health. 1997;18:341–78. Epub 1997/01/01. doi: 10.1146/annurev.publhealth.18.1.341 . 9143723 10.1146/annurev.publhealth.18.1.341

[5] McMahon J, Wanke C, Terrin N, Skinner S, Knox T. Poverty, hunger, education, and residential status impact survival in HIV. AIDS Behav. 2011;15(7):1503–11. doi: 10.1007/s10461-010-9759-z ; PubMed Central PMCID: PMCPMC3010417. 20632079 10.1007/s10461-010-9759-z PMC3010417

[6] Hogg RS, Strathdee SA, Craib KJ, O'Shaughnessy MV, Montaner JS, Schechter MT. Lower socioeconomic status and shorter survival following HIV infection. Lancet. 1994;344(8930):1120–4. Epub 1994/10/22. doi: 10.1016/S0140-6736(94)90631-9 . 7934494 10.1016/s0140-6736(94)90631-9

[7] Rapiti E, Porta D, Forastiere F, Fusco D, Perucci CA, Collaboration LAS. Socioeconomic status and survival of persons with AIDS before and after the introduction of highly active antiretroviral therapy. Epidemiology. 2000;11(5):496–501. doi: 10.1097/00001648-200009000-00003 WOS:000088854500003. 10955400 10.1097/00001648-200009000-00003

[8] Fordyce EJ, Singh TP, Nash D, Gallagher B, Forlenza S. Survival rates in NYC in the era of combination ART. J Acquir Immune Defic Syndr. 2002;30(1):111–8. Epub 2002/06/06. doi: 10.1097/00042560-200205010-00015 . 12048371 10.1097/00042560-200205010-00015

[9] Cunningham WE, Hays RD, Duan N, Andersen R, Nakazono TT, Bozzette SA, et al The effect of socioeconomic status on the survival of people receiving care for HIV infection in the United States. J Health Care Poor Underserved. 2005;16(4):655–76. doi: 10.1353/hpu.2005.0093 . 16311491 10.1353/hpu.2005.0093

[10] Pavlova-McCalla E, Trepka MJ, Ramirez G, Niyonsenga T. Socioeconomic Status and Survival of People with Human Immunodeficiency Virus Infection before and after the Introduction of Highly Active Antiretroviral Therapy: A Systematic Literature Review. J AIDS Clin Res. 2012;3(6). PubMed Central PMCID: PMCPMC3933225. 10.4172/2155-6113.1000163 PMC3933225 24575328

[11] McFarland W, Chen T, Hsu L, Schwarcz S, Katz M. Low socioeconomic status is associated with a higher rate of death in the era of highly active antiretroviral therapy, San Francisco. J Acquir Immune Defic Syndr. 2003;33(1):96–103. WOS:000182805400014. 12792361 10.1097/00126334-200305010-00014

[12] Shacham E, Nurutdinova D, Onen N, Stamm K, Overton ET. The interplay of sociodemographic factors on virologic suppression among a U.S. outpatient HIV clinic population. AIDS Patient Care STDS. 2010;24(4):229–35. doi: 10.1089/apc.2009.0275 ; PubMed Central PMCID: PMCPMC2864061. 20397898 10.1089/apc.2009.0275 PMC2864061

[13] Muthulingam D, Chin J, Hsu L, Scheer S, Schwarcz S. Disparities in engagement in care and viral suppression among persons with HIV. J Acquir Immune Defic Syndr. 2013;63(1):112–9. doi: 10.1097/QAI.0b013e3182894555 . 23392459 10.1097/QAI.0b013e3182894555

[14] World Health Organization. Cause-Specific Mortality Estimates for 2000–2012 Geneva2015 [cited 2016]. Available from: http://www.who.int/healthinfo/global_burden_disease/estimates/en/index1.html.

[15] UNAIDS. Gap Report Geneva: UNAIDS; 2014 [cited 2015 21 Dec]. Available from: http://www.unaids.org/en/resources/campaigns/2014gapreport.

[16] Barnighausen T, Hosegood V, Timaeus IM, Newell ML. The socioeconomic determinants of HIV incidence: evidence from a longitudinal, population-based study in rural South Africa. AIDS. 2007;21 Suppl 7(7):S29–38. doi: 10.1097/01.aids.0000300533.59483.95 ; PubMed Central PMCID: PMCPMC2847257. 18040162 10.1097/01.aids.0000300533.59483.95 PMC2847257

[17] Hargreaves JR. Socioeconomic status and risk of HIV infection in an urban population in Kenya. Trop Med Int Health. 2002;7(9):793–802. Epub 2002/09/13. doi: 10.1046/j.1365-3156.2002.00943.x . 12225512 10.1046/j.1365-3156.2002.00943.x

[18] Msisha WM, Kapiga SH, Earls F, Subramanian SV. Socioeconomic status and HIV seroprevalence in Tanzania: a counterintuitive relationship. Int J Epidemiol. 2008;37(6):1297–303. doi: 10.1093/ije/dyn186 ; PubMed Central PMCID: PMCPMC2638871. 18820319 10.1093/ije/dyn186 PMC2638871

[19] Fortson JG. The gradient in sub-Saharan Africa: socioeconomic status and HIV/AIDS. Demography. 2008;45(2):303–22. Epub 2008/07/11. doi: 10.1353/dem.0.0006 ; PubMed Central PMCID: PMC2831364. 18613483 10.1353/dem.0.0006 PMC2831364

[20] Fox A. The HIV-poverty thesis re-examined: poverty, wealth or inequality as a social determinant of HIV infection in sub-Saharan Africa? J Biosoc Sci. 2012;44(4). 10.1017/S0021932011000745 22273351

[21] Honge BL, Jespersen S, Nordentoft PB, Medina C, da Silva D, da Silva ZJ, et al Loss to follow-up occurs at all stages in the diagnostic and follow-up period among HIV-infected patients in Guinea-Bissau: a 7-year retrospective cohort study. BMJ Open. 2013;3(10):e003499 doi: 10.1136/bmjopen-2013-003499 ; PubMed Central PMCID: PMCPMC3808780. 24163204 10.1136/bmjopen-2013-003499 PMC3808780

[22] Geng EH, Odeny TA, Lyamuya RE, Nakiwogga-Muwanga A, Diero L, Bwana M, et al Estimation of mortality among HIV-infected people on antiretroviral treatment in east Africa: a sampling based approach in an observational, multisite, cohort study. Lancet HIV. 2015;2(3):E107–E16. doi: 10.1016/S2352-3018(15)00002-8 WOS:000363792000010. 26424542 10.1016/S2352-3018(15)00002-8 PMC4480204

[23] Mugglin C, Estill J, Wandeler G, Bender N, Egger M, Gsponer T, et al Loss to programme between HIV diagnosis and initiation of antiretroviral therapy in sub-Saharan Africa: systematic review and meta-analysis. Trop Med Int Health. 2012;17(12):1509–20. doi: 10.1111/j.1365-3156.2012.03089.x ; PubMed Central PMCID: PMCPMC3895621. 22994151 10.1111/j.1365-3156.2012.03089.x PMC3895621

[24] Bassett I, Wang B, Chetty S, Mazibuko M, Bearnot B, Giddy J, et al Loss to Care and Death Before Antiretroviral Therapy in Durban, South Africa. J Acquir Immune Defic Syndr. 2009;51(2). 10.1097/qai.0b013e3181a44ef2 PMC2747614 19504725

[25] Namusobya J, Semitala FC, Amanyire G, Kabami J, Chamie G, Bogere J, et al High retention in care among HIV-infected patients entering care with CD4 levels >350 cells/muL under routine program conditions in Uganda. Clin Infect Dis. 2013;57(9):1343–50. doi: 10.1093/cid/cit490 ; PubMed Central PMCID: PMCPMC3792723. 23899683 10.1093/cid/cit490 PMC3792723

[26] Cornell M, Myer L, Kaplan R, Bekker LG, Wood R. The impact of gender and income on survival and retention in a South African antiretroviral therapy programme. Trop Med Int Health. 2009;14(7):722–31. doi: 10.1111/j.1365-3156.2009.02290.x ; PubMed Central PMCID: PMCPMC2771267. 19413745 10.1111/j.1365-3156.2009.02290.x PMC2771267

[27] Birbeck GL, Kvalsund MP, Byers PA, Bradbury R, Mang'ombe C, Organek N, et al Neuropsychiatric and socioeconomic status impact antiretroviral adherence and mortality in rural Zambia. Am J Trop Med Hyg. 2011;85(4):782–9. doi: 10.4269/ajtmh.2011.11-0187 ; PubMed Central PMCID: PMCPMC3183792. 21976587 10.4269/ajtmh.2011.11-0187 PMC3183792

[28] Burkey MD, Weiser SD, Fehmie D, Alamo-Talisuna S, Sunday P, Nannyunja J, et al Socioeconomic determinants of mortality in HIV: evidence from a clinical cohort in Uganda. J Acquir Immune Defic Syndr. 2014;66(1):41–7. Epub 2014/01/01. doi: 10.1097/QAI.0000000000000094 ; PubMed Central PMCID: PMC3981890. 24378727 10.1097/QAI.0000000000000094 PMC3981890

[29] Mugusi FM, Mehta S, Villamor E, Urassa W, Saathoff E, Bosch RJ, et al Factors associated with mortality in HIV-infected and uninfected patients with pulmonary tuberculosis. BMC Public Health. 2009;9:409 doi: 10.1186/1471-2458-9-409 ; PubMed Central PMCID: PMCPMC2779816. 19909501 10.1186/1471-2458-9-409 PMC2779816

[30] Orrell C, Bangsberg D, Badri M, Wood R. Adherence is not a barrier to successful antiretroviral therapy in South Africa. AIDS. 2003;17(9). 10.1097/00002030-200306130-00011 12799558

[31] Talisuna-Alamo S, Colebunders R, Ouma J, Sunday P, Ekoru K, Laga M, et al Socioeconomic support reduces nonretention in a comprehensive, community-based antiretroviral therapy program in Uganda. J Acquir Immune Defic Syndr. 2012;59(4):e52–9. doi: 10.1097/QAI.0b013e318246e2aa ; PubMed Central PMCID: PMCPMC3887145. 22217680 10.1097/QAI.0b013e318246e2aa PMC3887145

[32] Richter LM, Lonnroth K, Desmond C, Jackson R, Jaramillo E, Weil D. Economic support to patients in HIV and TB grants in rounds 7 and 10 from the global fund to fight AIDS, tuberculosis and malaria. PLoS One. 2014;9(1):e86225 doi: 10.1371/journal.pone.0086225 ; PubMed Central PMCID: PMCPMC3904874. 24489702 10.1371/journal.pone.0086225 PMC3904874

[33] Karnofsky D, Abelmann W, Craver L, J. B. The use of the nitrogen mustards in the palliative treatment of carcinoma. Cancer. 1948;1(4).

[34] Castelnuovo B, Manabe YC, Kiragga A, Kamya M, Easterbrook P, Kambugu A. Cause-specific mortality and the contribution of immune reconstitution inflammatory syndrome in the first 3 years after antiretroviral therapy initiation in an urban African cohort. Clin Infect Dis. 2009;49(6):965–72. doi: 10.1086/605500 . 19673615 10.1086/605500

[35] Kamya MR, Mayanja-Kizza H, Kambugu A, Bakeera-Kitaka S, Semitala F, Mwebaze-Songa P, et al Predictors of long-term viral failure among ugandan children and adults treated with antiretroviral therapy. J Acquir Immune Defic Syndr. 2007;46(2):187–93. doi: 10.1097/QAI.0b013e31814278c0 . 17693883 10.1097/QAI.0b013e31814278c0

[36] Consolidated guidelines on the use of antiretroviral drugs for treating and preventing HIV infection: Recommendations for a public health approach World Health Organization, 2013. 24716260

[37] Antiretroviral therapy for HIV infection in adults and adolescents: Recommendations for a public health approach World Health Organization, 2010. 23741771

[38] Burkey MD, Weiser SD, Fehmie D, Alamo-Talisuna S, Sunday P, Nannyunja J, et al Socioeconomic Determinants of Mortality in HIV. JAIDS Journal of Acquired Immune Deficiency Syndromes. 2014;66(1):41–7. doi: 10.1097/QAI.0000000000000094 24378727 10.1097/QAI.0000000000000094 PMC3981890

[39] Phelan JC, Link BG, Tehranifar P. Social conditions as fundamental causes of health inequalities: theory, evidence, and policy implications. J Health Soc Behav. 2010;51 Suppl:S28–40. doi: 10.1177/0022146510383498 . 20943581 10.1177/0022146510383498

[40] Tuller DM, Bangsberg DR, Senkungu J, Ware NC, Emenyonu N, Weiser SD. Transportation costs impede sustained adherence and access to HAART in a clinic population in southwestern Uganda: a qualitative study. AIDS Behav. 2010;14(4):778–84. Epub 2009/03/14. doi: 10.1007/s10461-009-9533-2 ; PubMed Central PMCID: PMC2888948. 19283464 10.1007/s10461-009-9533-2 PMC2888948

[41] Castel AD, Kalmin MM, Hart RL, Young HA, Hays H, Benator D, et al Disparities in achieving and sustaining viral suppression among a large cohort of HIV-infected persons in care—Washington, DC. AIDS Care. 2016;28(11):1355–64. doi: 10.1080/09540121.2016.1189496 . 27297952 10.1080/09540121.2016.1189496 PMC5084086

