# Supplementary material for: Socioeconomic position and ten-year survival and virologic outcomes in a Ugandan HIV cohort receiving antiretroviral therapy
Source: PLoS One. 2017 Dec 15;12(12):e0189055. doi: 10.1371/journal.pone.0189055 (PMC5731768; doi:10.1371/journal.pone.0189055)
Supplement: S1 File — Table A. Risk factors for all-cause 10-year mortality in a Ugandan cohort with baseline AIDS receiving ART (N = 559). Table B. Risk factors for 10-year virologic treatment failure in a Ugandan cohort with baseline AIDS receiving ART (N = 472). (DOCX) [file pone.0189055.s001.docx]

S1 File

**Table A. Risk factors for all-cause 10-year mortality in a Ugandan cohort with baseline AIDS receiving ART (N=559).**

| **Baseline Category** | **Baseline Characteristics** | **Unadjusted Hazard Ratio (95% CI)** | ***P* value** | **Adjusted Hazard Ratio (95% CI)*** | ***P* value** |
| --- | --- | --- | --- | --- | --- |
| **Sex** | Men | 1.07 (0.74 - 1.56) | 0.70 | 1.07 (0.69-1.67) | 0.76 |
| **Age, years** | < 30 | 1.00 | Ref | 1.00 | Ref |
|  | 30 – 40 | 1.26 (0.77-2.06) | 0.35 | 1.97 (1.07-3.62) | 0.03 |
|  | > 40 | 1.83 (1.10-3.04) | 0.02 | 2.77 (1.46-5.24) | <0.01 |
| **Highest education level** | Primary | 1.00 | Ref |  |  |
|  | Secondary | 1.00 (0.69 - 1.45) | 0.99 |  |  |
|  | Tertiary | 1.08 (0.62 - 1.88) | 0.79 |  |  |
| **Employment status** | Unemployed | 1.00 | Ref | 1.00 | Ref |
|  | Self-employed | 0.58 (0.38-0.90) | 0.02 | 0.94 (0.54-1.62) | 0.82 |
|  | Organization/Govt | 0.43 (0.21-0.85) | 0.02 | 0.47 (0.16-1.35) | 0.16 |
|  | Privately employed | 0.58 (0.32-1.03) | 0.07 | 0.78 (0.35-1.77) | 0.56 |
| **Household income** | No regular income | 1.00 | Ref | 1.00 |  |
|  | Income <1 US$/day | 1.00 (0.67 - 1.50) | 0.99 | 0.92 (0.57-1.48) | 0.73 |
|  | Income >1 US$/day | 0.68 (0.43 - 1.07) | 0.10 | 0.86 (0.43-1.71) | 0.67 |
| **Physical housing** | Brick house | 1.00 | Ref |  |  |
|  | Mud house | 1.10 (0.76 - 1.58) | 0.62 |  |  |
| **Karnofsky Score** | Karnofsky ≥80 | 0.28 (0.20 - 0.40) | <0.001 | 0.43 (0.28-0.66) | <0.001 |
| **CD4 count/μL** | <100 | 1.00 | Ref | 1.00 | Ref |
|  | 100-200 | 0.48 (0.31 - 0.74) | <0.001 | 0.56 (0.34-0.92) | 0.02 |
|  | >200 | 0.61 (0.35 - 1.08) | 0.09 | 0.67 (0.34-1.30) | 0.24 |
| **Time-varying characteristics** | Karnofsky ≥80 | 0.05 (0.03 - 0.08) | <0.001 | 0.32 (0.22-0.47) | <0.001 |
|  | CD4 <100 | 1.00 | Ref | 1.00 | Ref |
|  | CD4 100-200 | 0.14 (0.08 - 0.24) | <0.001 | 0.36 (0.21-0.65) | <0.01 |
|  | CD4 >200 | 0.08 (0.04 - 0.13) | <0.001 | 0.37 (0.25-0.54) | <0.001 |

Hazard Ratio calculated via Cox proportional hazard model and *adjusted for sex, age, employment, income and baseline and time-varying Karnofsky and CD4.

**Table B. Risk factors for 10-year virologic treatment failure in a Ugandan cohort with baseline AIDS receiving ART (N=472)**

| **Baseline Category** | **Baseline Characteristics** | **Unadjusted Hazard Ratio (95% CI)** | ***P* value** | **Adjusted Hazard Ratio (95% CI)*** | ***P* value** |
| --- | --- | --- | --- | --- | --- |
| **Sex** | Men | 0.95 (0.63 - 1.44) | 0.81 | 0.81 (0.53-1.25) | 0.34 |
| **Age, years** | < 30 | 1.00 | Ref | 1.00 | Ref |
|  | 30 – 40 | 0.69 (0.45-1.07) | 0.10 | 0.73 (0.46-1.16) | 0.18 |
|  | > 40 | 0.61 (0.36-1.03) | 0.07 | 0.57 (0.33-0.99) | 0.05 |
| **Highest education level** | Primary | 1.00 | Ref | 1.00 | Ref |
|  | Secondary | 1.27 (0.85 - 1.88) | 0.24 | 1.22 (0.80-1.85) | 0.36 |
|  | Tertiary | 0.76 (0.37 - 1.55) | 0.45 | 0.67 (0.30-1.49) | 0.33 |
| **Employment status** | Unemployed | 1.00 | Ref | 1.00 | Ref |
|  | Self-employed | 0.74 (0.46-1.20) | 0.22 | 0.60 (0.37-0.98) | 0.04 |
|  | Organization/Govt | 0.98 (0.55-1.74) | 0.95 | 0.71 (0.36-1.41) | 0.33 |
|  | Privately employed | 1.03 (0.59-1.80) | 0.92 | 0.84 (0.42-1.70) | 0.63 |
| **Household income** | No regular income | 1.00 | Ref | 1.00 | Ref |
|  | Income <1 US$/day | 1.39 (0.85 - 2.27) | 0.19 | 1.81 (1.10-2.98) | 0.02 |
|  | Income >1 US$/day | 1.47 (0.90 - 2.39) | 0.12 | 2.33 (1.24-4.39) | 0.01 |
| **Physical housing** | Brick house | 1.00 | Ref |  |  |
|  | Mud house | 1.03 (0.69 - 1.54) | 0.89 |  |  |
| **Karnofsky Score** | Karnofsky ≥80 | 0.87 (0.58 - 1.32) | 0.52 |  |  |
| **CD4 count/μL** | <100 | 1.00 | Ref |  |  |
|  | 100-200 | 0.80 (0.52 - 1.21) | 0.29 |  |  |
|  | >200 | 0.84 (0.48 - 1.49) | 0.56 |  |  |
| **Time-varying characteristics** | Karnofsky ≥80 | 0.15 (0.08 - 0.30) | <0.001 | 0.46 (0.34-0.63) | <0.001 |
|  | CD4 <100 | 1.00 | Ref | 1.00 | Ref |
|  | CD4 100-200 | 0.34 (0.19 - 0.61) | <0.001 | 0.46 (0.34-0.63) | <0.001 |
|  | CD4 >200 | 0.07 (0.03 - 0.13) | <0.001 | 0.32 (0.25-0.42) | <0.001 |

Hazard Ratio calculated via Cox proportional hazard model and *adjusted for sex, age, education, employment, income and time-varying Karnofsky and CD4
